# High fecal carriage of extended Beta Lactamase producing *Enterobacteriaceae* among adult patients admitted in referral hospitals in Dar es Salaam, Tanzania

**DOI:** 10.1186/s12879-020-05272-4

**Published:** 2020-07-31

**Authors:** Upendo O. Kibwana, Mtebe Majigo, Doreen Kamori, Joel Manyahi

**Affiliations:** Department of Microbiology and Immunology, Muhimbili University of Health and Allied Sciences Dar es Salaam, P.O. Box 65001, Dar es Salaam, Tanzania

**Keywords:** ESBL producing pathogen, Enterobacteriaceae, Tanzania

## Abstract

**Background:**

Multi-drug resistance pathogens such as Extended-Spectrum Beta-Lactamase (ESBL) producing Enterobacteriaceae (ESBL-PE) are of great global health concern, since they are associated with increased morbidity and mortality. Even in the absence of infections caused by these pathogens, colonization is a great threat and can lead to cross transfer among hospitalized patients. To date data on carriage of these pathogens is still limited in Tanzania. Therefore, this study aimed to determine ESBL-PE fecal carriage rate and associated factors among hospitalized patients at Referral hospitals in Dar es Salaam.

**Methods:**

This was a cross sectional study conducted from May to July 2017 among patients admitted in three referral hospitals in Dar es Salaam, Tanzania. Rectal swabs were collected and screened for ESBL production using MacConkey agar supplemented with Ceftazidime 2 μg/ml. Phenotypic confirmation of ESBL-PE was done by double disk diffusion method. Statistical analysis was performed using Statistical Package for Social Sciences (SPPS) software version 20.

**Results:**

Of the 196 enrolled participants, 59.7% (117/196) were confirmed to carry ESBL-PE. Diarrheic patients (57/79) had statistically significant high prevalence of ESBL colonization compared to those without diarrhea (60/117) (*p* = 0.01). A total of 131 ESBL-PE were isolated from 117 patients, whereby, *Escherichia coli* accounted for 68.7%, *Klebsiella pneumoniae* 28.2% and *Citrobacter* species 0.8%. ESBL-PE carriage was significantly higher in patients with diarrhea compared to those without diarrhea (72% vs 53.1%, *p* = 0.01). Recent antibiotic use was independently associated with carriage of ESBL-PE (aOR 14.65, 95%CI 3.07–69.88, *p* = 0.01).

**Conclusions:**

High prevalence of fecal carriage of ESBL-PE was observed in patients admitted in tertiary hospitals in Dar es Salaam, Tanzania. The use of antibiotics was associated with carriage of ESBL producers among the study population.

## Background

Extended-spectrum β-lactamase producing Enterobacteriaceae (ESBL-PE) infections poses a unique challenge to healthcare, as it is associated with mortality and morbidity [1]. These pathogens are increasingly implicated as causes of both community and hospital-acquired infections but even in the absence of infection, colonization with Extended-spectrum β-lactamase (ESBL) producing bacteria is a reason for concern [2, 3]. In hospital settings, gastrointestinal carriage of ESBL, is the main reservoir of these organisms associated with high risk for developing self and cross infections [4].

ESBL-PE carriage and infections varies from different geographical locations, individual hospitals and different clinical conditions [5, 6]. A recent study from Ethiopia reported prevalence of ESBL-PE colonization to be 52% among hospitalized patients [7]; admission unit, number of beds and number of patients per room were reported as factors associated with carriage of ESBL-PE in that study. Several studies in East Africa have also documented high rates of ESBL-PE from clinical settings [8, 9].

In Tanzania, some few studies have been conducted, mostly focusing on infections caused by ESBL producers among different populations, from children to adults with different clinical conditions [10, 11], but little is known about carriage rate of these pathogens among hospitalized patients where cross contamination can occur. In addition, there is also limited information about carriage of ESBL producing pathogens among adult patients with diarrhea in the study area which is important to know since ESBL production can also be observed in pathogens causing diarrhea such as, diarrhogenic *E.coli* [12, 13]. Therefore, this study aimed to investigate ESBL-PE fecal carriage among hospitalized patients including those with diarrhea, and determine factors associated with their carriage in various referral hospitals in Dar es Salaam.

## Methods

### Study design and settings

This was a cross sectional study conducted from May to July 2017 in three hospitals, in Dar es Salaam, Tanzania. The study sites included Muhimbili National hospital (MNH) the main specialized tertiary hospital with 1500-bed capacity, admitting 150–180 patients per day of which 2% are due to diarrheal disease. In addition, Amana and Temeke, regional referral hospitals with 250–300 bed capacity each, admitting 50–70 patients per day of which 6–7% are due to diarrheal diseases.

### Study population, sample size and sampling procedure

A total of 196 adult patients aged 18 years and above who were admitted in medical wards, Intensive Care Unit (ICU) and isolation wards for more than 24 h were randomly selected and consecutively enrolled in this study.

### Data collection

Structured questionnaires were used to collect study participants’ clinical and demographic information. Information recorded included; age, sex, admission unit, diarrhea status, antibiotic use in the past 3 months, hospitalization history in the past 3 months, history of invasive procedures in the past 3 months and co-morbidities.

### Specimen collection

Trained nurses collected rectal swabs from consented participants and immediately put in Cary Blair transport media. The specimens were transported in a cool box with ice packs to Microbiology and Immunology Bacteriology research laboratory at Muhimbili University of Health and Allied Sciences (MUHAS) for processing.

### Laboratory investigations

In the laboratory, rectal swabs were cultured immediately on MacConkey agar supplemented with Ceftazidime 2 μg/ml and incubated aerobically at 37 °C for 24 h as a screening test for ESBL-PE [14]. Isolated bacteria were identified based on colonial morphology, Gram staining and a set of conventional biochemical tests which included, Indole, Citrate, Sulphur Indole Motility (SIM) as described elsewhere [15] and API 20E tests.

Isolated organisms in screening test were potentially considered as ESBL-PE; however, they were further confirmed by double disk diffusion method [16]. Briefly, both ceftazidime (30 μg) and cefotaxime (30 μg), alone and in combination with clavulanate (10 μg) were placed into inoculated Muller Hinton Agar (MHA) plate with test organism and incubated at 37 °C aerobically for 18 h. The zones of inhibition were observed and interpreted according to Clinical and Laboratory Standards Institute (CLSI) 2015 guidelines. ESBL-PE was confirmed when there was ≥5 mm increase in a zone diameter for either antimicrobial agent tested in combination with clavulanate versus when tested alone [16]. *Klebsiella pneumoniae* ATCC 700603 was used as a positive control.

### Antibiotic susceptibility test

The confirmed ESBL-PE isolates were tested for antimicrobial susceptibility using Kirby-Bauer disk diffusion method according to CLSI guidelines [16]. Briefly, homogenous colonial suspensions were prepared using 3–5 colonies from a pure culture comparable to 0.5 McFarland turbidity standard. Standardized suspensions were inoculated on MHA, and then incubated at 37 °C aerobically for 24 h. The zones of inhibition were interpreted according to CLSI guidelines. *E. coli* ATCC 25922 was used as a positive control organism. The disks used included: chloramphenicol (20 μg), gentamicin (10 μg), ciprofloxacin (5 μg), trimethoprim/sulfamethoxazole (1.25/23.75 μg), tetracycline (30 μg) (Oxoid, UK).

### Data analysis

Data analysis was performed using statistical package for social science (SPSS) version 20. Categorical variables were summarized in a form of frequencies and percentages. Fisher’s exact test was employed to compare the associated factors for ESBL-PE. *P*-value < 0.05 was considered as statistically significant. Binary simple logistic regression was first performed to determine the factors associated with ESBL-PE followed by multivariable regression model to determine factors independently associated with ESBL-PE carriage. Adjusted odds ratio was used to describe strength of association at 95% confidence intervals. *P*-value < 0.05 was considered as statistically significant.

## Results

### Description of study participants

A total of 196 inpatients were enrolled, with age range from 18 to 62 years and median age of 25 (IQR: 18–30) years. Half of the study participants were aged between 18 and 27 years, and those above 57 years were the least (0.5%). Most of participants 61.7% (121/196) were females and Medical ward unit contributed majority of patients accounting for 78.6%. Nearly half of participants (46.9%) had history of previous antibiotic use in the past 3 months and 18.4% had history of invasive procedures in the past 3 months majority of them (55.6%) being catheterization. Seventy-nine patients (40.3%) had diarrhea and 36 patients (18.4%) were HIV positive (Table 1).

**Table 1:** Socio-demographics and clinical Characteristics of study participants

Variables	Frequency/Median	Percentage (%)
**Median age 25**		
**Age group in years**		
18–27	98	50.0
28–37	68	35.0
38–47	22	11.0
> 47	8	4.0
**Sex**		
Female	121	67.7
Male	75	35.3
**Study Site**		
Temeke	166	84.7
Amana	20	10.2
MNH	10	5.1
**Admission Unit**		
Medical ward	154	78.6
Isolation Unit	35	17.9
ICU	7	3.5
**Diarrhea Status**		
Yes	79	40.3
No	117	50.7
**Antibiotic use in the past 3 months**		
Yes	92	46.9
No	104	53.1
**Hospitalization in the past 3 months**		
Yes	70	35.8
No	126	64.2
**Invasive procedure in past 3 months**		
Yes	36	18.4
No	160	81.6
**Type of Invasive procedure**		
Catheterization	20	55.6
Surgery	16	44.4
**HIV status**		
Positive	36	18.4
Negative	160	81.6

### Distribution of ESBL producing Enterobacteriaceae carriage

Screening and phenotypic testing of isolates obtained from rectal swab specimen for ESBL-PE revealed that the overall fecal carriage of ESBL-PE was 59.7% (117/196). Majority of study participants colonized by ESBL-PE aged between 38 and 47 years (68%) and there was no significant difference in carriage between the age groups. Diarrheic patients 57 (72%) had higher colonization rate than those without diarrhea and the difference was statistically significant (*p* = 0.01). There was also a significant high carriage in patients admitted at Amana hospital (80%), compared to Temeke hospital and Muhimbili National hospital (*p* = 0.004). Further analysis stratification revealed that higher carriage rate in patients admitted in medical wards (61%) compared to ICU (57.1%) and isolation units (60%) (*p* = 0.01) (Table 2).

**Table 2:** Distribution of ESBL producing Enterobacteriaceae among study participants

Characteristics	***N*** = 196	Proportion of ESBL-PE carriage	
		n (%)	*p*-value
**Overall 59.7%**			
**Gender**			
Male	75	43 (57.3)	0.780
Female	121	74 (61.2)	
**Age groups (years)**			
18–27	98	56 (57.1)	
28–37	68	42 (61.8)	
38–47	22	15 (68.2)	0.592
> 47	8	4 (50.0)	
**Study Site**			
Temeke	166	95 (57)	
Amana	20	16 (80)	0.004
MNH	10	6 (60)	
**Study Unit**			
Medical Ward	154	92 (61)	
Isolation Ward	35	21 (60)	0.001
ICU	7	4 (57.1)	
**Diarrhea Status**			
Yes	79	57 (72)	0.001
No	117	60 (53.1)	

### Bacteria isolates and antimicrobial resistance pattern of ESBL-PE

Among 131 bacteria isolates confirmed as ESBL producers, *E coli* (68.7%) and *K. pneumoniae* (28.2%) were the most frequent isolated bacteria. All isolated ESBL-PE showed high co-resistance to antibiotics tested (Table 3). Highest resistance totrimethoprim/sulfamethoxazole (> 85%) was observed in all isolates. *E. coli* displayed high rates of resistance to Gentamycin (82.1%), Ciprofloxacin (66.3%, Chloramphenicol (77.9%) trimethoprim/sulfamethoxazole/ (85.3%) and Tetracycline (84.2%).

**Table 3:** Antimicrobial resistance pattern of isolated ESBL-PE

Antibiotic	***E. coli*** % (***N*** = 90)	***K. pneumoniae*** % (***N*** = 37)	***K. oxytoca % (N = 3)***
Gn	82.1	82.4	66.7
Cip	66.3	73.5	66.7
C	77.9	67.6	66.7
Sxt	85.3	97.1	100
Tet	84.2	91.2	100

*Gn* gentamicin, *Cip* ciprofloxacin, *C* chloramphenicol, *Sxt* trimethoprim/sulfamethoxazole/, *Tet* Tetracycline

### Factors associated with carriage of ESBL-PE

Analysis was performed to determine the factors associated with carriage of ESBL-PE. Patients exposed to antibiotics up to 3 months prior to hospitalization were significantly associated with colonization by ESBL-PE (cOR 10.7, 95% CI, 2.90–39.42, *p* = 0.00). There was no significant association between carriage of ESBL-PE and patients with history of previous hospital admission in the past 3 months as well as HIV status. On multivariable analysis, previous antibiotic use for the past 3 months was found to be independently associated with the carriage of ESBL-PE (aOR 14.65, 95% CI 3.07–69.88, *p* = 0.01) (Table 4). An additional excel file describes all results in more detail (see additional file 1).

**Table 4:** Univariate and Multivariate analysis of factors associated with fecal carriage of ESBL producing Enterobacteriaceae

Characteristics	***N*** = 129	n (%) =117	Proportion of ESBL carriage			
			Univariate		Multivariate	
			cOR (95%)	***p*** value	aOR (95%)	***p*** value
**Gender**			1^a^			
Male	48	43 (89.6)				
Female	81	74 (91.4)	1.23 (0.37–4.11)	0.74		
**Age group in years**						
	116	105 (90.5)	1^a^			
	13	12 (92.3)	1.26 (0.15–10.60)	0.83		
**Antibiotic use in the past 3 months**						
No	16	10 (62.5)	1^a^			
Yes	113	107 (94.7)	10.7 (2.90–39.42)	0.00	14.65 (3.07–69.88)	0.01
**Hospitalization in the past 3 months**						
No	87	78 (89.7)	1^a^			
Yes	42	39 (92.9)	1.50 (0.38–5.86)	0.56		
**Invasive procedures in the past 3 months**						
No	95	87 (88.4)	1^a^			
Surgery	13	12 (92.3)	1.26 (0.15–10.61)	0.83		
Catheterization	21	18 (85.7)	1.833 (0.45–7.43)	0.4		
**Chronic disease**						
No	120	90 (75.0)	1^a^			
Yes (HIV/AIDS, PTB, Diabetes)	29	27 (92.3)	1.30 (0.27–6.28)	0.75		

N: Total number of patients subjected to confirmatory ESBL test, n: number of patients confirmed to carry ESBL producing pathogens, 1^a^: reference category, *cOR* crude odds ratio, *aOR* adjusted odds ratio, *HIV* Human Immunodeficiency Virus, *AIDS* Acquired Immunodeficiency Syndrome, *PTB* Pulmonary Tuberculosis

## Discussion

In the present study we demonstrate high carriage rate of ESBL producing pathogens among hospitalized patients which is associated with previous antibiotic use. We further demonstrate high rates among patients with diarrhea compared to patients admitted with other conditions. The overall carriage rate (57.9%) in our study is comparable to findings from hospitalized adults in Ethiopia (52%) and Rwanda (50%) [7, 17]. However, our finding is slightly higher compared to that reported among hospitalized patients by Sabrina et al. in Dar es Salaam, Tanzania [11]. Potential reasons for the observed difference include several factors such as; different ESBL detection techniques and specimen used, whereas in a study by Sabrina et al. they used urine specimen and ESBL-E test strip for ESBL detection. Furthermore the carriage rate in our study is also higher compared to that reported among hospitalized children in Dar es Salaam, Tanzania [18]. This difference may be accounted for by the difference in study population, whereby the participants in that study were children versus adults in our study. Being adults, our study participants are likely to have been exposed to risk factors for carriage of ESBL-PE, like previous hospitalization and antibiotic use including self-prescription compared to children. In Uganda, a much higher colonization rate of 62% has been reported [19]. The variation between our study and this study may be due to differences in study population, specimen taken, clinical conditions of the participants, factors which are known to cause variation in ESBL-PE colonization patterns as documented by others [20, 21].

Among the ESBL-PE colonized patients in our study, those with diarrhea had higher carriage rate compared to those without diarrhea. These results are similar to what others have reported [6, 22]. However, in a study done among children by Tellevic and his colleagues, children hospitalized due to diarrhea had lower carriage rate compared to those hospitalized due to other diseases, probably because the latter group included children with many diverse conditions [18]. The pattern observed in our study can be explained by the fact that diarrhea cause changes in the gut microbiota which provides conducive environment for exchange of resistance genes between inter and intra species including the indigenous organisms [23].

In an attempt to investigate factors associated with ESBL-PE carriage, previous antibiotic use in the past 3 months was identified as a significant factor associated with carriage of ESBL. Our findings are in agreement with previous studies done in Rwanda [17, 24]. This observation might be reflecting the result of irrational use of these antibiotic agents in the study population that eventually may lead to high selection pressure of resistant bacteria.

In line with reports from Sudan [25] and Mali [26], *Escherichia coli* was the most predominant ESBL producer isolated in our study. Cross-resistance of ESBLs to other drug classes such as aminoglycosides and fluoroquinolones has been previously documented [27–29]. Findings from this study corroborates with that, as it indicated a high co-resistance pattern to those antibiotic classes in all tested isolates. This pattern is however higher than findings from Ethiopia [7]. This may be explained by the fact that the plasmids carrying genes for ESBL production which was observed to be high in this study was also carrying co- resistance genes for other antibiotics. Highest resistance of antibiotics was observed in trimethoprim/sulfamethoxazole/ and tetracycline in all isolated ESBL-PE, these findings are consistent with reports from one study in Uganda [30]. The high resistance pattern observed in this study could reflect frequent use of over the counter medicine and self-medication practices in the study settings. This result indicates the need for guided treatment following culture results. A limitation of this study was a failure to perform molecular tests for characterization of ESBL genes from isolated pathogens.

## Conclusion

This study revealed high prevalence of fecal carriage of ESBL-PE among hospitalized patients especially among those with diarrhea. Previous antibiotic use was associated with carriage of ESBL-PE. ESBL producing isolates expressed high resistance to other commonly used antibiotics.

## Supplementary information

**Additional file 1.** Raw data for ESBL-PE among adult patients in Dar es Salaam Tanzania. Additional file 1 is an excel spreadsheet with the data obtained from admitted adult patients in three different hospitals in Dar es salaam Tanzania. All provided data was analyzed using SPSS software and generated results reported in this manuscript.

## Data Availability

All relevant data generated and analyzed during this study are included in this manuscript.

## Abbreviations

AIDS: Acquired Immunodeficiency Syndrome
AST: Antimicrobial Susceptibility Testing
ATCC: American Type Culture Collection
CLSI: Clinical Laboratory Standard Institute
ESBL: Extended Spectrum Beta Lactamase
ESBL-PE: Extended Spectrum Beta Lactamase producing Enterobacteriaceae
HIV: Human Immunodeficiency Virus
MHA: Muller Hinton Agar
MUHAS: Muhimbili University of Health and Allied Sciences
SPSS: Statistical Package for Social Sciences
UK: United Kingdom
